# Tenth Annual Report of the State Lunatic Hospital, at Worcester, Massachusetts, December, 1842

**Published:** 1843-08

**Authors:** 


### REVIEWS.

---

Art. VIII.—*Tenth Annual Report of the State Lunatic Hospital, at Worcester, Massachusetts*, December, 1842. Boston, 1843: 8vo., p. 115.

2. *Fourth Annual Report of the Directors and Superintendent of the Ohio Lunatic Asylum, Columbus*, December, 1842: 8vo., p. 88.

3. *Twenty-sixth Annual Report of the state of the Asylum for the relief of persons deprived of the use of their reason.* Philadelphia, 1843; 8vo., p. 27.

4. *The Annual Report of the Physician and Superintendent of the Eastern Asylum, in the city of Williamsburg, Virginia, for* 1842. Richmond, 1843: 8vo., p. 38.

The first report before us is from the pen of Dr. Woodward, the gentleman who has had charge of the Hospital since its establishment. Dr. Woodward has rendered himself well and very favorably known to the profession, through his Annual Reports; and has elicited much well merited praise in his own State, for the great amount of good that he has done, in connection with the institution of which he has charge. Since its opening, in 1833, fifteen hundred and fifty-seven persons have been admitted into it, laboring under the various forms of insanity; in the time thirteen hundred and nineteen have been discharged, of whom six hundred and seventy-six were restored to reason; and of the remainder about two fifths were improved; about the same number not improved; and one-fifth either died or eloped. By a somewhat troublesome calculation (which, we protest, the Doctor ought to have made himself, especially as it exhibits a good condition of things, when we take into consideration the necessary accumulation of old and difficult cases in Asylums for the insane), we find that the per cent. of recoveries on the whole number of patients who enjoyed the benefits of the Hospital during each successive year was:

| 1833 | 1834 | 1835 | 1836 | 1837 | 1838 | 1839 | 1840 | 1841 | 1842 |
|---|---|---|---|---|---|---|---|---|---|
| 16.3 | 27.4 | 21.5 | 23.6 | 22,5 | 20.9 | 20.1 | 20.9 | 20.5 | 20.4 |

By similar calculations we find that the:

| | 1833 | 1834 | 1835 | 1836 | 1837 | 1838 | 1839 | 1840 | 1841 | 1842 |
|---|---|---|---|---|---|---|---|---|---|---|
| Per cent. of the discharged improved on the whole number in the Hospital in the course of each year, was: - - - - - - - - | 4.5 | 9.4 | 9.5 | 6.9 | 7.5 | 6.6 | 7.3 | 7.4 | 9.0 | 5.9 |
| Per cent. of recoveries on all admitted during each year, was: - - - - - | 16.3 | 53.7 | 46.0 | 46.4 | 41.0 | 42.9 | 49.1 | 50.6 | 50.3 | 44.4 |
| Per cent. of recoveries of males on the males admitted: - - - - - - - - | 13.5 | 48.5 | 52.9 | 48.4 | 39.3 | 46.8 | 40.0 | 37.0 | 50.6 | 41.1 |
| Per cent. of recoveries of females on the females admitted: - - - - - | 21.0 | 60.7 | 40.3 | 44.0 | 43.2 | 48.2 | 48.4 | 62.0 | 50.0 | 48.3 |

The per cent. of all the recoveries of males in ten years on all the males admitted in that time, was 40.7. And the per cent. of all the recoveries of females, in the same time, on all the females admitted, was 46.3.

There is perhaps no question, connected with insanity, of greater importance than the comparative curability of it, at the different periods of the disease. The statistics of all hospitals contain abundant evidence of its far greater curability, if subjected to treatment within the first year; and superintendents are constantly urging in their reports, the importance of sending patients early to asylums, where they can undoubtedly be much better treated than at home. At the hospital whose report we are examining, the per cent. of the discharged cured on the number of the recent cases* ad-

* By *recent* cases is meant those whose insanity was of less duration than one year, at the time of admission; and by *old* cases, those of longer duration than a year.

mitted (for the year 1842), was 64.2, whilst the per cent. of the old cases was only 20.2. Out of the admissions for the same year too, only one of the recent cases was considered incurable; whilst a per cent. of 56.1 of the old cases was discharged as incurable and harmless; and 15.6 discharged as incurable and dangerous. In the whole ten years, there was a per cent. of 88.9 of the recent cases cured, or considered curable; 47.4 per cent. of those who had been insane from one to five years; and only 10.8 of those who had been insane from five to fifteen years.

The report contains also a table which exhibits a favorable state of things in Massachusetts, in reference to the cause of temperance as it bears upon insanity. A proportion of 14.4, of all who have been received into the hospital in ten years, was produced by intemperance. Of the first fourth of the patients, a per cent. of 20.8 owed their attack to this cause; of the second fourth 13.8 per cent; of the third fourth 13.1; and of the last 9.7; showing a progressive and rapid diminution of insanity, as temperance advanced. Whilst however temperance has tended thus plainly and rapidly to diminish insanity, a new and somewhat fruitful source has made its appearance; we mean *Millerism.* In reference to this subject, Dr. Woodward makes the following excellent remarks: and most fully do we concur with him in the sentiments expressed. "Some new views of religious truth," says he, "have recently disturbed many persons who have deep solicitude for their future well-being, and have brought a number of patients under our care. Some of these views are greatly calculated to alarm those who entertain them, and I greatly fear that, for some months to come, this agitation of the public mind may, in this and other communities, add many to the list of the insane.

"Religion, in any view of it, is a solemn subject for contemplation. No individual can feel indifferent to it who has a rational mind, and feels his responsibility to God for the actions of his life. But it is particularly desirable that all consideration of it should be calm and dispassionate, that we should *live it* in our several spheres of duty rather than seek new dogmas which distract the mind, and unfit it for the high responsibilities of this life, or for suitable preparation for the elevated pleasures of a future existence.

"The Bible itself would rarely make a man insane; its promises counterbalance its denunciations, and its plain and simple instruction shows most clearly the way to pardon and to peace. It is human dogmas and new-fangled doctrines, promulgated by the ignorant and misguided, which are at present distracting the public mind, loosening the cords which bind society together, and, without chart or compass, set mankind forth in search of a heavenly inheritance. When the settled principles of religious faith and hope are discarded, when fanaticism predominates, and the established forms of religious worship are abandoned, then it is that the minds of the weak and excitable are distracted and made insane; then it is that the effort to reach something indefinable and untangible, overpowers the intellect, and often breaks it down and destroys it. This is not religion, but its counterfeit—a base moral currency, unsafe, and worse than useless in its influence, corrupting instead of reforming its victims, and levelling, rather than elevating the moral and religious standard of the community in which it circulates."

*Ohio Lunatic Asylum.*—According to the history given by the Directors, the Territorial and State Legislation in Ohio, upon the subject of idiots and lunatics, consisted in several acts passed at different times from 1792 to 1815. These acts, in accordance with the prevailing notions of the times, provided merely for a trial by jury to determine the question of idiocy or lunacy in an individual, and for their "close confinement." And, shocking as it would now appear to us, this "confinement" was to be in the county jail, "unless his friends should give sufficient bond for his safe keeping;" and "if committed to jail, the county commissioners were required "carefully to examine him, and if, in their opinion, medical aid should be requisite, to employ a skillful physician." County commissioners to determine the curability, the possibility of improvement, &c., &c., &c., of a case of insanity! Bless us! What a commentary on Diagnosis!

Pursuing, very briefly, the history of this subject in Ohio, and passing over this benighted period which continued unchanged for more than twenty years (for we feel inclined to cast a mantle of forgetfulness over the past, and turn to a contemplation of the present, or indulge a lingering look at the beautiful perspective of the near and distant future), we learn that, stimulated by an "eloquent memorial" from the Medical Convention of Ohio, the Legislature in the winter of 1835 passed an act, making the necessary provisions for the establishment of her Lunatic Asylum. In March, 1838, a beautiful site having been purchased in the suburbs of Columbus, and the building nearly completed, an act was passed for the government of the asylum; and in the following November, it went into operation. In October, about a month before it went into operation, we ourselves spent an hour or two in the building and grounds; and a vivid impression do we retain of the adaptation of the buildings to the comfort and convenience of the patients and officers; and of the susceptibility of the grounds to be made into such a place as our imagination painted for the health, pleasure, occupation, and amusements of the insane. Whether such improvements have been made, we are not able to say from personal observation, and the report does not inform us.

Since the institution went into operation, four hundred and eight patients have enjoyed its benefits; and, in the whole period, two hundred and sixty-five applications for admission have been rejected for want of room. Based on this, and some other grounds, the Directors with great propriety urge upon the Legislature the importance of making an appropriation for the erection of additional buildings, for the reception of patients.

The legitimate object of reports from Lunatic Asylums in general, is (so far as the medical profession is concerned) to exhibit statistics, whereby we may learn the nature and tendency of insanity, whether to cure, continuance, or death, and the effect of treatment upon it. And the object of reports from particular asylums should, of course, be to exhibit their own condition in relation to these same matters. Accordingly we find on page thirty-three of the report before us, a "recapitulation," made up from a long tabular view showing the statistics of the Asylum from its institution. After giving all the facts, as to the admissions, discharges, recoveries, deaths, &c., the superintendent then, to save his readers the trouble of the calculation, exhibits the per centage of recoveries. And by a superficial examination, we would readily infer that the per centage as given by him, is indicative of the effect of the treatment adopted in the Asylum of which he has charge. But a little scrutiny led us to detect a very essential error in his mode of calculation, by which (if the object be to show the effect of treatment) very incorrect conclusions would be induced. The method of calculation employed exhibits the *per cent.* of "recoveries," on the cases which have been *discharged* from the Asylum; whereas, if the object be that stated above, the *per cent.* should, of course, be upon the number *admitted* into the Asylum, and who have been subjected to treatment. This is obvious, for the reason that the *number* of "*recoveries*" remaining the same, the per centage would be materially affected, as you compare them (the recoveries) with the whole number of any class of patients who have been admitted, or merely with the number of the same class discharged. For instance, the report exhibits:

| | | |
|---|---|---|
| Whole number of patients admitted (since the Asylum was instituted), - - | 408 | |
| Old cases, - - - - - - - | 237 | 408 |
| Recent do. (less duration than one year), | 171 | |
| Whole number discharged, - - - | 266 | |

| | | |
|---|---|---|
| Old cases, - - - - - - - | 123 | 266 |
| Recent do., - - - - - - - | 143 | |
| Whole number of *old cases* recovered, - | 43 | 165 |
| Whole number of recent cases recovered, | 122 | |

Taking these facts as the basis of his calculation, and comparing the "recoveries" with the *discharges*, the superintendent gives us:

| | |
|---|---|
| "Per cent. of recoveries on all the cases discharged, - - - - - - - - | 62.03 |
| "Per cent. of recoveries on all the old cases discharged, - - - - - - - | 34.95 |
| "Per cent. of recoveries on all the recent cases discharged, - - - - - - - - | 85.31" |

Whereas, taking the same data, if we compare the "recoveries" with the *admissions*, we have:

| | |
|---|---|
| Per cent. of recoveries on all the cases admitted, - - - - - - - - | 40.44 |
| Per cent. of recoveries of old cases on all the old cases admitted, - - - - - - | 18.14 |
| Per cent. of recoveries of recent cases on all the recent cases admitted, - - - - - | 71.34 |

We find too that the same error has crept into the calculation for the hospital year just ended (1842). But from the coincidence of there having been the same number of admissions and discharges, it does not affect the result in reference to all the cases; and the difference between the admissions and discharges of both the old and recent being so small, the result is not very materially affected.

| | | |
|---|---|---|
| Number admitted the present year (1842). | Old cases, | 30 |
| | Recent, | 35=65 |
| Number discharged (same year). | Old cases, | 31 |
| | Recent, | 34=65 |
| Number of discharged recovered (same year). | Old cases, | 13 |
| | Recent, | 28=41 |

Pursuing the same faulty mode of calculation the report exhibits:

1st. "Per cent. of recoveries on all the cases discharged the present year, - - - - 63.07

2d. "Per cent. of recoveries on the entire number of old cases discharged the present year, including thirteen discharged by the directors for want of room. - - - - - - - 41.93

3d. "Per cent. of recoveries on all the recent cases discharged the present year. - - - 82.35"

Whereas, comparing the recoveries with the *admissions* gives for:

1st. - - - - - - - - - 63.07
2d. - - - - - - - - - 43.33
3d. - - - - - - - - - 80.00

We feel no disposition whatever to attach blame to the superintendent, Dr. Awl, or to ascribe false statements to him; for we have made all the calculations, and find his statements of per centage to be correct according to his mode of calculation. Neither do we wish to attribute to him the purpose of endeavoring to exhibit the institution of which he has charge in a false light; but only to call attention to the fact that the per centage as given in the report, does not afford an index of the effect of treatment of insanity in the Ohio Asylum. It may be urged that it is right to compare the "recoveries" with the "discharges;" since that, some who have been "admitted and subjected to treatment" may be still in the hospital, and with a chance for recovery. But, this can be claimed only for those who come in towards the close of a year for which a particular report may be made; whilst it could not with any propriety whatever be asserted, if we are examining into the results of a series of years.

We have endeavored, by referring to the reports from several other Asylums, to ascertain the general custom in reference to this matter; but most of those which we have in our reach contain but very little upon the subject of per centage, except the one from the Massachusetts Asylum, which we have just noticed; and that does not contain the necessary data to allow us to make a calculation for ourselves, except in one or two particulars, wherein we find that Dr. Woodward employed the same *mode of calculation* that we have objected to above. But even if at Worcester, and all the other Asylums in the country, this mode of reckoning per centage is employed, it by no means convinces us of the propriety of it; on the other hand, it would more loudly call for a free expression of opinion, convinced as we are that the method is not a fair one, and decidedly calculated to produce erroneous impressions.

The third report is from the institution generally known as the Friend's Asylum for the insane, near Frankford. This Asylum has been in operation twenty-six years; and in that time six hundred and sixty persons have been admitted as patients. Of this number two hundred and seventy-three have been restored; seventy-one much improved; and ninety-two died. At the end of the hospital year for 1841, there were fifty-eight patients remaining in the Asylum. During the present year twenty-five have been admitted; thirty-two discharged and five died; of the discharged ten were restored, six much improved, four improved, and twelve stationary.

Of the fifty-eight remaining at the end of the year, only eighteen were placed under treatment; the balance being deemed incurable. And of the twenty-five admitted the present year only twenty-two were placed under treatment, for the same reason. So that during the year, forty have been under treatment. Out of this number nineteen were recent cases, nine of whom were restored, three convalescent, one much improved, four stationary, one died, and one just received; leaving just fifty *per cent.* of the recent cases cured, with some probability of the per centage being increased by the continuance of treatment with the remaining cases.

The remaining twenty-one, who were subjected to treatment during the year, were all old cases; and only four of them were restored. Giving us nineteen *per cent.* of cures for this class.

*Eastern Asylum, Williamsburg, Virginia.*—This venerable Asylum was established in 1769; and was like all other asylums of its day, the recipient of patients laboring under a disease universally esteemed almost incurable. They were all then rather places for the safe-keeping and confinement of the insane; for it was seldom that patients were ever taken to hospitals until they had become unmanageable by their friends, and often not until after they had been confined a year or more in a county jail. Placed in a hospital under such circumstances, common sense would teach that there was but little chance of recovery; and modern statistics fully confirm the opinion. How little encouragement then to physicians, having charge of such patients, to make any adequate efforts for their restoration! On the other hand, again, the very small number who were restored and the large number who remained inmates of hospitals for years and years, under close confinement, and severe and often cruel treatment, gave but little inducement to their friends to place them there.

How changed now the scene! Then, confinement and severity were the great principles of treatment; and were carried into effect by methods, which, if pictured to the mental vision, could scarcely be comprehended. Now, freedom, kindness, sympathy, occupation, companionship, and amusements, are the great elements of treatment. How changed the results too! Then, an extremely small per centage recovered their reason; and they, probably, rather despite, than in consequence of the treatment. Now, a large majority speedily return to gladden the hearts of their friends, when they can be induced to send them early to suitable institutions. We of the present day can make estimates by figures of the change which has been wrought, and fancy indeed that we form a tolerable conception of it. But let us imagine if we can, what would have been the feelings of an intelligent man of those days, happily gone by forever, who had been accustomed to see the insane in their cells, raving under the aggravation of chains and confinement, or given up to despair from the absence of all sympathy, what, we say, would have been his feelings, if the veil that hides the future could have been drawn aside, and there presented to his vision the interior and grounds of a well regulated Insane Asylum of the present day, with its numerous inmates receiving humane and kind treatment at the hands of the superintendent and keeepers, and pursuing various occupations and amusements; and eight or nine tenths rapidly recovering from the worst of human maladies. If one drop of philanthropic blood coursed through his veins, verily his rapture could not have been greater if the "crystal bar of Eden" had moved from before his eager eyes; and, if the doctrine of letting every body provide for himself and his own had taken possession of his nature, we suppose that the contemplation would have given a rebuke to him not lightly to be borne, and have even stirred his heart to a more kindly consideration for his fellow men.

This train of reflection has been suggested by the report of the Superintendent of this old Asylum, which furnishes in itself an almost complete history of insanity. Established under the Colonial Government, it was the recipient for the insane of an extended country; and even under the then mistaken plan of management, doubtless greatly meliorated their condition. During the revolution, the British soldiery wanting the house for a barracks turned loose its inmates to roam at large, and seek shelter and sustenance wherever their benighted intellects might carry them: an act more Vandal-like, if possible, than the burning of our capitol during the late war. In 1785 it was again refitted for its original purposes, and has continued to be used as an Insane Hospital ever since.

Owing to the circumstance of there being ample provision made by Virginia for all her insane, patients are never discharged from the Asylum that we are noticing except by recovery or death; and, consequently, during the long period of its existence, a great many old and incurable cases have accumulated. Up to the 1st of July, 1841, when the present Superintendent, Dr. John M. Galt, took charge of the institution, one hundred and nine patients, most of them of this character, were in the Asylum. During the first six months following this, fourteen were admitted, eleven discharged, and fifteen died. Of the eleven discharged, nine were cured, and one so much improved that he soon after recovered. During the first year of the present Superintendent's management, thirteen recent cases were admitted; twelve of whom were cured and discharged by the end of the year 1842; and the remaining one died. This furnishes a per centage of 92.3 of recoveries from this class of cases; a larger proportion, we believe, than has ever been given by any other institution in our country. And indeed, as the one who died labored rather under delirium tremens than insanity, the Superintendent puts in a claim for excluding his case from the calculation; which would give 100 per cent. for the recoveries. We are not furnished with the necessary data by which we could ascertain the result of treatment in old cases.

An interesting feature, in the history of this old Asylum, is that the medical attendance upon it has descended from the grand-father to the father and the son. But it was not until the son came into office that the duties of physician and Superintendent were merged in the same individual, and the modern method of managing patients was adopted. The results have proved eminently successful and creditable to the young man, to whom this important trust has been committed; and we wish him a long, useful, and happy life.

G. W. B.

P. S. Since this article went to press we have discovered some errors in our calculations on page 115. The reader will please make the following corrections: 3d column of first table, for 21.5 read 22.5; 4th column, for 23.6 read 23.7. Second table, 3d column: for 9.5 read 9.9; 4th column, for 6.9 read 7.5; 6th column, for 46.8 read 48.9, and for 48.2 read 38.3; 7th column, for 7.3 read 7.4; for 49.1 read 44.6; 8th column, for 37.0 read 37.3. 16th line from bottom, for 40.7 read 46.4.

